# Novel Machine Learning HIV Intervention for Sexual and Gender Minority Young People Who Have Sex With Men (uTECH): Protocol for a Randomized Comparison Trial

**DOI:** 10.2196/58448

**Published:** 2024-08-20

**Authors:** Ian W Holloway, Elizabeth S C Wu, Callisto Boka, Nina Young, Chenglin Hong, Kimberly Fuentes, Kimmo Kärkkäinen, Mehrab Beikzadeh, Alexandra Avendaño, Juan C Jauregui, Aileen Zhang, Lalaine Sevillano, Colin Fyfe, Cal D Brisbin, Raiza M Beltran, Luisita Cordero, Jeffrey T Parsons, Majid Sarrafzadeh

**Affiliations:** 1 Department of Social Welfare UCLA Luskin School of Public Affairs University of California, Los Angeles Los Angeles, CA United States; 2 Department of Epidemiology UCLA Fielding School of Public Health University of California, Los Angeles Los Angeles, CA United States; 3 Department of Computer Science UCLA Samueli School Of Engineering University of California, Los Angeles Los Angeles, CA United States; 4 School of Social Work Portland State University Portland, OR United States; 5 Mindful Designs Teaneck, NJ United States

**Keywords:** HIV, mobile health, mHealth, text messaging, machine learning, mobile app, sexual and gender minority, Young Men’s Health Project, YMHP, motivational interviewing, substance use, harm reduction, mobile phone

## Abstract

**Background:**

Sexual and gender minority (SGM) young people are disproportionately affected by HIV in the United States, and substance use is a major driver of new infections. People who use web-based venues to meet sex partners are more likely to report substance use, sexual risk behaviors, and sexually transmitted infections. To our knowledge, no machine learning (ML) interventions have been developed that use web-based and digital technologies to inform and personalize HIV and substance use prevention efforts for SGM young people.

**Objective:**

This study aims to test the acceptability, appropriateness, and feasibility of the uTECH intervention, a SMS text messaging intervention using an ML algorithm to promote HIV prevention and substance use harm reduction among SGM people aged 18 to 29 years who have sex with men. This intervention will be compared to the Young Men’s Health Project (YMHP) alone, an existing Centers for Disease Control and Prevention best evidence intervention for young SGM people, which consists of 4 motivational interviewing–based counseling sessions. The YMHP condition will receive YMHP sessions and will be compared to the uTECH+YMHP condition, which includes YMHP sessions as well as uTECH SMS text messages.

**Methods:**

In a study funded by the National Institutes of Health, we will recruit and enroll SGM participants (aged 18-29 years) in the United States (N=330) to participate in a 12-month, 2-arm randomized comparison trial. All participants will receive 4 counseling sessions conducted over Zoom (Zoom Video Communications, Inc) with a master’s-level social worker. Participants in the uTECH+YMHP condition will receive curated SMS text messages informed by an ML algorithm that seek to promote HIV and substance use risk reduction strategies as well as undergoing YMHP counseling. We hypothesize that the uTECH+YMHP intervention will be considered acceptable, appropriate, and feasible to most participants. We also hypothesize that participants in the combined condition will experience enhanced and more durable reductions in substance use and sexual risk behaviors compared to participants receiving YMHP alone. Appropriate statistical methods, models, and procedures will be selected to evaluate primary hypotheses and behavioral health outcomes in both intervention conditions using an α<.05 significance level, including comparison tests, tests of fixed effects, and growth curve modeling.

**Results:**

This study was funded in August 2019. As of June 2024, all participants have been enrolled. Data analysis has commenced, and expected results will be published in the fall of 2025.

**Conclusions:**

This study aims to develop and test the acceptability, appropriateness, and feasibility of uTECH, a novel approach to reduce HIV risk and substance use among SGM young adults.

**Trial Registration:**

ClinicalTrials.gov NCT04710901; https://clinicaltrials.gov/study/NCT04710901

**International Registered Report Identifier (IRRID):**

DERR1-10.2196/58448

## Introduction

### Background

Sexual and gender minority (SGM) young adults are disproportionately affected by HIV in the United States, and substance use is a major driver of new infections. Among men who have sex with men (MSM) in the United States, the lifetime risk of receiving an HIV diagnosis is 1 in 6, compared to 1 in 524 among heterosexual men [1]. According to the 2022 Centers for Disease Control and Prevention (CDC) *HIV Diagnoses, Deaths, and Prevalence* report, approximately 70% of new HIV diagnoses were attributable to male-to-male sexual contact [2]. Young MSM are at particularly high risk for HIV and sexually transmitted infections (STIs). The CDC in the same report found that young people aged 13 to 34 years accounted for approximately half of all new HIV diagnoses in the United States in 2022. Transgender women in the United States have also been found to be at an increased risk for HIV [3]. A 2021 CDC report found that 4 in 10 transgender women surveyed in 7 major US cities are living with HIV [4]. While transgender women made up the large majority of new HIV diagnoses within transgender and gender nonbinary populations at 87%, HIV incidence among all these identities substantially rose between 2018 and 2022, with a staggering 136% rise in diagnoses among gender nonbinary persons, 33% in transgender men, and 24% in transgender women [2].

In the United States, evidence suggests that substance use is associated with condomless anal sex and increased numbers of sexual partners [5-9], both of which may increase one’s risk of HIV infection. MSM have been shown to be 3 to 4 times more likely to report substance use compared to the general population [10-12]. HIV infection among transgender women has also been associated with polysubstance use and sexual risk behaviors [13]. Compared to the populations of cisgender men and transgender women, little has been established in existing literature that examines the effects of sexual risk and substance use behaviors contributing to HIV and STIs in transgender men and gender nonbinary individuals. Our work uses the definition of *transgender* as individuals whose gender identity differs from their assigned sex at birth [14] and *gender nonbinary* as an umbrella term for individuals whose gender identity is neither man nor woman, encompassing a variety of experiences including but not limited to identification with multiple genders, no gender, or a gender that changes over time [15].

Lesbian, gay, and bisexual adults in the United States are approximately twice as likely to use web-based dating sites and apps compared to their heterosexual counterparts [16]. People who use web-based venues to meet sexual partners are more likely to report substance use, sexual risk behaviors, and STIs [17-19]. Geosocial networking apps, such as Grindr (Grindr, Inc) and Tinder (Match Group, Inc), use GPS to facilitate more efficient dating and networking with others based on proximity, which in turn may increase the likelihood of STI acquisition in specific geographical areas [20]. Research has also indicated that some MSM, transgender women, and gender nonbinary people use digital technologies to search for and intentionally use illicit drugs before or during sex to sustain, enhance, disinhibit, or facilitate the sexual experience, which may contribute to increased risk of HIV and other STIs through substance-induced disinhibition and increased length of sexual sessions [21,22].

Although web-based and social media data have been used by researchers in interdisciplinary fields for a range of health-related applications [23-25], to our knowledge, no interventions have been developed that use machine learning (ML) to inform HIV prevention and substance use harm reduction efforts for both MSM and other SGM populations. Previous research suggests the feasibility of applying social media and web-based data mining as a public health surveillance strategy [26-29]. ML, a branch of computer science that uses statistical and optimization tools to “learn” patterns and trends from large quantities of data, can be used to identify or predict new patterns or trends [30]. This approach has been used to detect and predict other health-related outcomes, such as preterm births [31], health care monitoring of patients with chronic illnesses [32], and suicide prevention [33]. On an individual level, insights gleaned from web-based and social media use patterns can inform cost-effective, scalable interventions that are highly personalized and delivered via technology (ie, tailored messaging and outreach from health workers).

The use of web-based and social media data mining and ML approaches within SGM populations for HIV prevention and substance use harm reduction has important implications for public health. Our team previously developed and tested a social media data mining program that correctly identified HIV risk and amphetamine and methamphetamine use using only social media data [34]. This system was refined and tested for this intervention trial.

There are few “best” rated evidence-based risk reduction interventions (EBRRIs) in the CDC Compendium of Prevention Research Synthesis for young adult, HIV-negative MSM. To our knowledge, there is currently 1 “best” rated EBRRI for young transgender women [35] and no “best” rated EBRRIs for other SGM populations, such as transgender men and gender nonbinary people. One of the few “best” rated EBRRIs for young gay and bisexual men is the Young Men’s Health Project (YMHP), which is a manualized 4-session intervention that uses motivational interviewing (MI) and problem-solving skills to provide personalized feedback for reducing risk behaviors and promoting HIV prevention skills such as using HIV pre-exposure prophylaxis (PrEP) [36]. It is also the only intervention to show significant effects in reducing both condomless anal sex and substance use. YMHP has also been effectively used in remote delivery [37]. The YMHP intervention component, MI, is a proven approach in HIV or STI prevention for many populations [38,39]. However, few research studies have tested the effectiveness of MI for risk reduction with other SGM people, including transgender women, transgender men, and gender nonbinary young people [40,41].

### This Study

We hypothesize that the uTECH+YMHP intervention will be considered acceptable, appropriate, and feasible by most participants. Furthermore, we hypothesize that participants receiving the combined uTECH+YMHP intervention will show greater and more sustained reductions in substance use and sexual risk behaviors compared to those receiving the YMHP intervention alone. Specifically, we anticipate that the integration of ML-informed SMS text messages will enhance the efficacy of YMHP counseling methods by providing personalized, timely, and contextually relevant support, ultimately leading to improved health outcomes in this sample of SGM young adults.

## Methods

### Overview

This protocol was created following the SPIRIT (Standard Protocol Items: Recommendations for Interventional Trials) reporting guidelines (Multimedia Appendix 1) [42]. The proposed study is a randomized comparison trial to evaluate the acceptability, appropriateness, and feasibility of uTECH, which is a text-based risk reduction intervention that leverages a data mining and ML algorithm to provide personalized prevention messaging to users. uTECH will be compared to an existing best-evidence MI intervention (YMHP). Participants will be randomly assigned to one of two conditions: (1) uTECH+YMHP (trial intervention+existing intervention) or (2) YMHP only (existing intervention only). Those in the uTECH+YMHP condition will receive 4 YMHP sessions with a trained master’s-level social worker and tailored SMS text messages. Those in the YMHP arm will only receive 4 YMHP sessions. Participants in both conditions will either contribute location-tracking and keylogger smartphone data through an app called “eWellness” (version 1.0.24) or provide data downloads from their most-used, web-based dating and social media accounts. These data will be used to inform and prime the ML algorithm that will be used to make predictions and generate tailored SMS text messages for the uTECH+YMHP condition. The overarching goal of this study is to evaluate the acceptability, appropriateness, and feasibility of uTECH in conjunction with YMHP (condition 1) compared to YMHP alone (condition 2). This study was registered with ClinicalTrials.gov under NCT04710901.

### Target Population

This study aims to recruit 330 participants. Half (n=165, 50%) of the participants will be randomized to receive uTECH+YMHP (condition 1), and the remaining half (n=165, 50%) will be randomly assigned to receive YMHP only (condition 2).

To be eligible for the study, participants must be aged 18 to 29 years, identify as SGM, have had anal or oral sex with a man (defined by the participant’s interpretation) in the past 3 months, have used substances (such as alcohol, marijuana, amyl nitrate [poppers], methamphetamines, heroin, cocaine, and ecstasy) in the past 3 months, have had sex while using substances in the past 3 months, have a negative or unknown HIV status, and have used a dating app to meet sexual or substance use partners in the past 3 months. Participants must also own a smartphone, reside in the United States, be willing to participate in a 12-month study, and be able to provide informed consent. Participants that use an Android smartphone as their primary device must consent to install the eWellness app on their phone to remotely collect keystroke and geolocation data throughout the duration of the study. Participants who use an iOS (Apple Inc) smartphone must provide the study team with at least 1 set of data from both their primary dating app and their most-used social media account every 6 months. Failing to meet all the stated criteria would disqualify individuals from enrollment in the study. Exclusion criteria were conceived to build upon existing literature for HIV prevention in SGM populations while also expanding gender criteria to be inclusive of transgender and gender nonbinary individuals who have sex with men. Our study population aims to capture the identities of young adult users of queer-centered dating apps and websites such as Grindr, Scruff (Perry Street Software, Inc), and Sniffies (Sniffies, LLC) who may be at heightened risk of HIV and STI transmission [17-19], particularly in the context of substance use [5-9].

### eWellness App Development

The mobile app, eWellness, was developed during phase 1 of this study and uses the AWARE framework [43], which is an open-source context instrumentation that uses smartphone sensor instruments to infer, log, and share mobile information for application developers and researchers. The AWARE framework has been used in numerous earlier eHealth studies, for example, to predict depression and anxiety [44,45], progression of Parkinson disease [46], or alcohol use events [47]. We developed eWellness using the AWARE framework and dashboard to record keylogging data (data entered into the user’s preferred smartphone keyboard), which are remotely downloaded to secure databases daily. eWellness is also enhanced with a user-friendly interface that provides several “tabs” of information, including a study timeline and incentive chart; a brief survey; and an interactive Google-based map of nearby HIV and STI testing locations, risk reduction supportive services, needle exchanges, and other harm reduction and substance use treatment services.

The data collected through the eWellness app are used to refine and “teach” the ML algorithm to make predictions that can be used to inform the uTECH intervention. During the 12-month design process, we developed a working version of the app and added additional features as they were developed. We recruited SGM people (n=20) aged >18 years who used Android phones and used websites and apps to seek sexual or substance use partners. These phase-1 participants downloaded the app, which collected data that were used by the study team to further refine the ML algorithm.

Throughout the 12-month study, we also conducted semistructured interviews with the participants to obtain feedback and preferences on possible app functions. Interviewers followed a questionnaire developed by the principal investigator and research staff. Data from these phase-1 interviews were analyzed using rapid qualitative analysis [48], and insights were used to refine and finalize the app in preparation for phase 2.

During phase 2, the eWellness app will be installed by participants who use Android smartphones. Once installed, the app will collect data continuously for 12 months. The app collects the following data: keystrokes (letters and numbers that the user types into their smartphone keyboard), the time and date of information that is typed into the keyboard, the name of the app used when typing in an app, and geolocation. The app does not collect the following data: any data that are not typed directly into the smartphone keyboard, photos or camera data, accelerometer data, video, drop-down menu selections, barometer, audio or microphone data, battery data, text-to-speech data, Bluetooth data, address book contacts, gyroscope data, screen time tracking data, gravity sensor data, incoming SMS text messages, magnetometer data, phone logs, proximity sensor data, installation logs, scheduler data, network sensor availability, temperature data, rotation sensor data, Wi-Fi data, telephony data, or time zone data.

### Social Networking App Downloads

Participants who use iOS smartphones will be asked to provide social networking app account downloads from their most frequently used web-based dating apps, such as Grindr or Tinder, and social media accounts, such as Twitter or X (X Corp), Instagram (Meta Platforms, Inc), Reddit (Reddit, Inc), and Facebook (Meta Platforms, Inc), to send to the research team. The data requests will occur at baseline and at 6-month and 12-month follow-up interviews. The exact data request process varies for each app but generally involves the user going into the app settings and requesting a data download that will be sent to the participant’s email address. The participant then downloads their data and emails the files to an encrypted email address managed by the research team.

Data requests may include but are not limited to text-based content of social media posts, profile information, chats and messages they send to others, like or viewing history, advertisement history, and background activity (such as time spent on the app, geolocation, and account settings).

### YMHP Intervention

The YMHP intervention consists of four 1-hour counseling sessions that use MI as a collaborative, goal-oriented approach of communication to discuss how participants manage sexual health and substance use in their daily lives. The goal of the YMHP counseling sessions is to explore these issues while supporting participants in making informed decisions for their health [36]. The sessions will be conducted by doctoral students trained in YMHP, holding master’s degrees in social work, and supervised by a licensed clinical social worker.

There is a wealth of evidence demonstrating the effectiveness of MI for behavioral change [49]. MI is rooted in the principles of engaging, focusing, evoking, and planning. The practice and implementation of MI use deep listening skills, effective reflections, and strategies for responding to resistance. Engaging involves building rapport and creating a sense of alliance through approaching participants with an attitude of curiosity, compassion, and understanding. Focusing involves exploring the participant’s view of their situation and selecting a target behavior for change. Although the main target behaviors are sexual health and substance use, understanding the participant’s priorities and goals is essential. Evoking is the act of finding out the participant’s own reasons for change and brainstorming how to reach those goals. Evoking can be achieved using open-ended questions and the Ask-Tell-Ask method [50]. Planning involves the creation of a course of action and the anticipation of obstacles. The transition into the planning stage should only be done when the participant is ready and willing to do so.

For this study, YMHP will be tailored and used with young adults across the SGM spectrum and will include gay and bisexual men as well as transgender women, transgender men, and gender nonbinary individuals who have sex with men, reflecting our study population.

### The uTECH Intervention

Participants randomized to receive the uTECH intervention (uTECH+YMHP) will receive SMS text messages that use the Information-Motivation-Behavior Skills (IMB) model [51] to improve substance use harm reduction and promote safer sex practices. The IMB model was developed to use information, motivation, and behavioral skills to promote HIV-related behavior change, positing that when groups at risk for HIV are well informed, motivated to act on their knowledge, and have the necessary behavioral skills to seek out and initiate change, they will successfully overcome obstacles to make behavior change. The IMB model is effective in enhancing protective health behaviors such as adherence to interventions or reduction of risk behaviors with people living with HIV or AIDS [52-54] and people who use substances [55-57].

Staff will follow the IMB framework to generate content for the SMS text messages, a process that will be informed by an extensive review of public health messaging, scientific literature, and validated survey instruments tailored for specific subgroups that will be recruited for this study. The groups include people who inject drugs, people who do not inject drugs, people who are on PrEP, people who are not on PrEP, transgender and nonbinary people who are on PrEP, and transgender and nonbinary people who are not on PrEP. Each participant will be assigned to 1 of the above subgroups based on their answers in their baseline interview. The SMS text messages will be sent to participants using Twilio (version 8.0.0; Twilio Inc) [58], a web-based communications service that can be programmed to send and receive SMS text messages. Approximately 2 SMS text messages will be scheduled per week, so that each participant will receive 100 text messages over the course of 12 months.

To obtain feedback, participants will be asked to respond to each SMS text message. Participants will be compensated US $1 per SMS text message to which they reply. Each SMS text message also includes an opt-out option to comply with cell carrier antispam policies. The feedback from participants will also be used to enhance the ML algorithm in preparation for fully automated SMS text message generation and assignment (Table 1).

**Table 1:** Text message content examples from the uTECH intervention using the Information-Motivation-Behavior Skills (IMB) model.

	PrEP^a^ initiation	PrEP adherence	Sexual risk reduction	Substance use harm reduction
Information	“PrEP reduces the risk of getting HIV from sex by approximately 99%—if taken on an ongoing basis before sex and continued after sex. It is also called Truvada or Descovy.”	“Did you know? The Food and Drug Administration approved injectable PrEP, which is an option for people who are tired of taking a pill every day. It is an injection given every 2 months. Find out more at: https://www.fda.gov/news-events/press-announcements/fda-approves-first-injectable-treatment-hiv-pre-exposure-prevention.”	“If you had unprotected sex, you can take PEP (postexposure prophylaxis) up to 72 hours after possible exposure to prevent HIV. Watch a video to learn more: https://www.youtube.com/​watch?v=Yu82TFo6j94&featu”	“Here is a tip for staying hydrated while drinking: drink 1 glass of water for every alcoholic beverage you have. This is known as the 1-for-1 rule and may help moderate your drinking and lessen the chance of a hangover.”
Motivation	“Did you know that studies show taking PrEP lowers HIV-related anxiety? Are you less anxious about HIV when you are on PrEP?”	“Did you know that studies show taking PrEP lowers HIV-related anxiety? Are you less anxious about getting HIV when you are on PrEP?”	“Nervous about getting STI^b^ testing? It is safer to get tested and know the results. Here is how others overcame their concerns about STI testing: https://www.youtube.com/​watch?v=rN5d_XQKRxg.”	“If you ever inject with a friend, you could consider doing so at a SIS^c^. SIS refers to places where people who use injectable, but illegal, opioids such as heroin can do so without fear of overdose, prosecution, or spreading disease. You can try looking up sites in your area to find them or contact a member of the research team for assistance.”
Behavior	“Reply with the number of the statement that applies to you: 1. I have talked to a medical provider about getting PrEP but have not yet started taking it. 2. I have already talked to a medical provider about getting PrEP and have begun taking it. 3. I have not talked to a medical provider about getting PrEP and have not taken it yet.”	“Reply with the number of the statement that applies to you: Have you at any point considered stopping PrEP? 1. No, I am happy with PrEP and expect to continue taking it. 2. No, I am neither happy nor unhappy with PrEP, it just does not seem like an option to stop PrEP use. 3. Yes, but I changed my mind and expect to continue taking it. 4. Yes, and I did stop use for a period of time. Since then, I have resumed and plan to continue taking it. 5. Yes, and I am planning on discontinuing use soon.”	“Reply with the number of the statement that applies to you: 1. In the past month, I have gotten tested for HIV or ≥1 STIs. 2. In the past month, I have not gotten tested, but I have booked an appointment or set a date to get tested in the near future. 3. In the past month, I have not gotten tested and currently do not have an appointment or plan to do so.”	“Reply with the number of the statement that applies to you: Have you ever trusted someone to supervise you (or trip-sit) as you inject for safety purposes? 1. Yes, always. 2. Yes, but only once or a few times. 3. Yes, but I would not do it again. 4. No, but I would like to in the future. 5. No, I do not feel comfortable being supervised.”

^a^PrEP: pre-exposure prophylaxis.

^b^STI: sexually transmitted infection.

^c^SIS: safe injection site.

### Recruitment

#### Web-Based Outreach

Participants will be recruited through web-based outreach methods including targeted advertising on social media sites (ie, Meta, Grindr, Craigslist [Craigslist Inc], and Reddit), with a focus on Facebook Pages and Groups, or forums that may attract SGM individuals who use substances. The research team will also contact community organizations and prominent users with high follower counts to broaden recruitment efforts on social media sites such as OnlyFans (OnlyFans Ltd) and Instagram.

#### In-Person Outreach

The research team will distribute recruitment materials such as business cards, flyers, and palm cards at events that may attract SGM individuals who use substances; sexual health clinics; lesbian, gay, bisexual, transgender, and queer centers; substance use treatment centers; nonprofit organizations; and retail businesses. Staff will reach out to community-based organizations and retail businesses by phone and email, offering to mail recruitment materials to display at their locations to broaden recruitment nationwide.

#### Referral Program

Both participants and nonparticipants will be given the opportunity to refer others to the study and receive compensation for their referrals. Those interested in the referral program will be given information about the study, a sample message to send to others for referral purposes, and a link to sign up as a referrer so that the study staff can compensate them. Participants will be offered the opportunity to receive physical recruitment materials by mail (flyers and palm cards) to refer people within their social networks. Referrers will receive US $20 sent to their payment method of choice (Venmo [PayPal Holdings Inc], Cashapp [Block Inc], Paypal [PayPal Holdings Inc], or Amazon [Amazon Inc] e-gift card) for every person referred into the study that completes their baseline appointment (up to 5 people total).

### Enrollment Process

#### Screening

All participants will first complete a web-based screener survey on Qualtrics (Qualtrics International Inc), a web-based survey platform [59]. The screener will provide information about the study and ask the participant questions to determine eligibility and their willingness to provide keystroke and geolocation data (Android users) or social media data (iOS users). If the potential participant meets initial eligibility criteria, the survey will use branching logic to show additional screens for the individual to provide contact information, including email address and phone number.

Research staff will review screener responses daily and closely examine responses to check for fraudulent or false eligibility. Qualtrics collects users’ geolocation information and IP addresses, which will be used to input into a third-party fraud-checking website, such as Scamalytics [60]. The information we receive from Qualtrics will be checked using such websites to determine whether their phone number is a voice-over IP (or noncellular, internet) number or whether their IP address has been blacklisted for fraudulent activity or uses virtual private network, which would enable non-US users to bypass geolocation-based restrictions. “Eligible” screened participants who use a voice-over IP or virtual private network or come from an IP address with high fraudulent activity will be coded as “fraud” and will not be contacted by study staff.

Those who submit screeners that appear legitimate and who are eligible according to study criteria will be contacted by research staff via phone call, email, or SMS text message to schedule an onboarding. Participants will be provided a link to Calendly [61], a web-based calendar scheduling service, to pick a time for their onboarding session and will receive automated reminders for their appointments once booked. All appointments will take place over the Zoom videoconferencing platform. The interviewer will confirm that the participant will be in a safe and private setting to talk about confidential information for the duration of the call before beginning the session.

#### Randomization

All eligible participants will have their information entered into a password-protected and encrypted database that is only accessible by Health Insurance Portability and Accountability Act–certified and institutional review board–approved research staff. Alongside contact information, all participants will be assigned a participant ID number and computer-generated randomized assignment to 1 of 2 intervention groups: uTECH+YMHP or YMHP only. This randomization will be done before onboarding so that the onboarding study staff will know which type of onboarding will be required.

### Onboarding Procedures

#### Preparing for the Session

Before the participant joins their onboarding Zoom call, the interviewer will review the crisis management protocol, open up the appropriate forms for the participant’s mobile device (iOS or Android), and review the participant tracking sheet to ensure that they have the participant’s information from the screener. The interviewer will join the Zoom virtual conference room 5 to 10 minutes in advance of the participant and will have their camera on for the duration of the call. The participant will not be required to have their camera on. When the participant joins the Zoom call, the interviewer will confirm with the participant that they have 60 to 90 minutes available for the appointment and confirm that the participant is in a safe and private setting.

#### Consenting (All Participants)

The interviewer will open the appropriate consent form for the participant’s phone type (Android or iOS) and study condition (uTECH+YMHP or YMHP only), which is a survey hosted on Qualtrics, and share their screen on Zoom so that the participant can view it simultaneously. The interviewer will remind the participant that they will receive a copy of the document via email at the end of the call. The interviewer will input the participant ID at the top of the page and review study details while regularly asking the participant if they have any questions. The consent form includes a detailed explanation of all study requirements and how their data will be protected. At the end of the consent form, the interviewer will ask if the participant consents to the study. If affirmative, the interviewer will check the box for “I agree to participate in the study,” provide their initials on the survey, and submit the consent survey. If the participant does not consent, then the interviewer will check the box for “I decline to participate in the study,” provide their initials on the document, submit the survey, thank the participant for their time, and end the call. The consent process will take approximately 25 minutes. If the participant consents to participate, then the onboarding session will continue.

#### Installation of the eWellness App (Android Users Only)

Participants who use Android smartphones will receive step-by-step instructions on how to install the eWellness app (version 1.0.24, released January 2023) on their primary smartphone, where it will remain active and collect data for 12 months. The interviewer will send the participant a short link that they can type in their Android internet browser and download the app. The interviewer will guide the participant through the app download and setup process and troubleshoot any technical issues that may arise. If the interviewer or the participant runs into technical difficulties that cannot be resolved at the time of the interview, the interviewer will continue onto the baseline survey and make a note that they will have to complete the app download within a week’s time after consulting with the research team. If the app is compatible with the device and successfully downloads, the interviewer will continue to guide the participant in completing the in-app Wellness Survey. This process will take approximately 15 minutes.

#### Wellness Survey (iOS Users Only)

The Wellness Survey is a Qualtrics survey version of the in-app Wellness Survey that Android users complete. The participant receives a link to this survey through SMS text message, and the interviewer will instruct the participant to complete this short survey. This process will take approximately 5 minutes.

#### PrEP Verification (All Participants)

If the participant endorses current PrEP use, the interviewer will ask the participant to upload a photo of their prescription bottle to the eWellness app, which is routed to a Qualtrics survey that is encrypted with Transport Layer Security for all transmitted data. Participants may also choose to block or cover any personal information they do not wish to share with researchers. This process will take approximately 5 minutes.

#### Social Networking App Download Requests (iOS Users Only)

Participants who use iOS smartphones will receive step-by-step instructions on how to request social networking app downloads from their most frequently used web-based dating apps (such as Grindr or Tinder) and social media accounts (such as Twitter or X, Instagram, Reddit, and Facebook) to send to the research team. The study staff will describe the process specific to each dating app or social media app and either email the instructions to the participant or walk the participant through the process during the Zoom meeting. The participant will then need to download their data and email the files to an encrypted email address managed by the research team. This process will take approximately 10 minutes.

#### Baseline Interview (All Participants)

The interviewer will share their screen to display the baseline survey, which is hosted on the Qualtrics platform, and includes a series of questions about demographics, intimate partner history, sexual health, substance use, intimate partner violence, vaccine hesitancy, effects of COVID-19, and eHealth mobile app tracking and notification preferences. The survey also asks the participants for their preferred payment information. After all questions have been answered, the interviewer will stop sharing their screen and submit the baseline survey. This process will take approximately 30 minutes.

#### YMHP Scheduling (All Participants)

The interviewer will schedule the participant with their first YMHP session through Calendly, a scheduling website, with an interventionist who is not the baseline interviewer. The participant’s first session will be scheduled approximately 1 week after the onboarding and baseline survey (week 1). The interventionist and the participant will receive a calendar invite reminder for this session. Subsequent sessions are scheduled between the interventionist and the participant and will take place in week 2, month 1, and month 2 after onboarding. The scheduling process will take approximately 5 minutes.

#### Closing Out the Onboarding (All Participants)

The interviewer will open the “Locator form” to document the participants’ preferred forms of contact, their phone type, and how they heard about the study. The interviewer will remind the participant of the follow-up surveys at 3, 6, 9, and 12 months; their first YMHP session (if applicable); and when to expect payment (approximately 3-5 business days after each data collection point or YMHP session). This process will take approximately 10 minutes.

#### Postonboarding Activities (All Participants)

Immediately after ending the onboarding session with the participant, the interviewer will download the materials completed by the participant and upload them to a secure, password-protected cloud-based database (such as Box). Afterward, to ensure confidentiality, the interviewer will delete all materials from their computer. The interviewer will also update the participant’s information in a tracking database and send a thank you email to the participant, which will include a copy of the study’s consent form and the University of California, Los Angeles (UCLA) Office of Human Research Protection Program participant bill of rights [62], which details protections for participants within the consent and participation process. This process will take approximately 30 minutes.

#### STI or HIV Testing (All Participants)

Participants will be offered the option of sending STI or HIV test results to the study team at their baseline and 6-month appointment dates. While testing is optional, those who do elect to send results will be compensated US $30 at both points (for a total of US $60) for providing test results for HIV, syphilis, chlamydia, and gonorrhea. Participants will be instructed to send their results in the form of a scanned document, screenshot, or PDF to an encrypted email managed by the study team. All identifying information in the file containing results will be redacted by the research team before being saved in the study’s password-protected, encrypted, cloud-based database. When test results are received, participants will be reminded of their compensation and encouraged to reach out to research staff or their YMHP interventionist if they would like further information or resources.

If requested, participants will be provided HIV, syphilis, gonorrhea, or chlamydia prevention and treatment resources. They will also be asked if they have a health care provider who they could contact for treatment. If they do not, they will be offered a list of sexual health clinics or providers in their area. In addition, participants will be offered a partner notification service to anonymously notify recent sexual partners of a possible STI exposure.

#### YMHP Sessions (All Participants)

##### Overview

**Figure 1:**
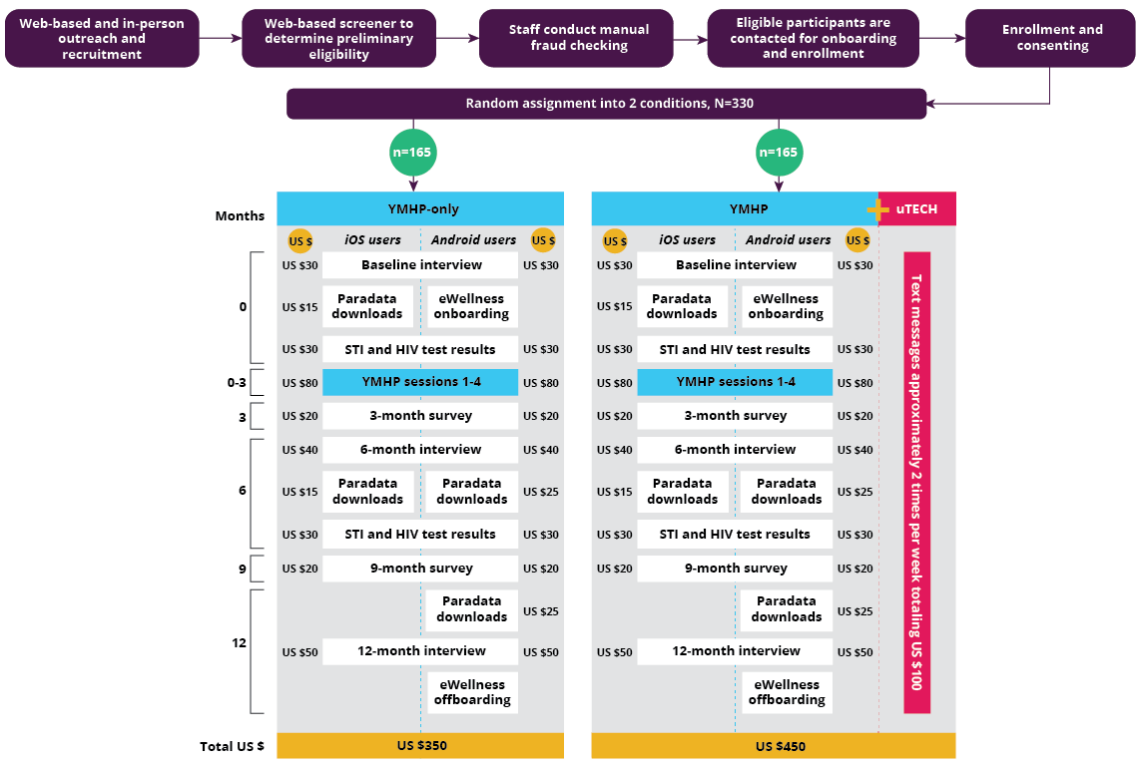
uTECH participation overview flowchart. STI: sexually transmitted infection; YMHP: Young Men’s Health Project.

YMHP intervention sessions will be held over Zoom, a videoconferencing platform. Session 1 will take place during week 1 (following onboarding), session 2 during week 2, session 3 during month 1, and session 4 during month 2 (Figure 1). All sessions will be audio recorded for intervention fidelity and ongoing intervention training and clinical support. No video or participant names will be captured for the recording. Participants will be asked to change their Zoom name to their participant ID before recording.

##### Session 1: Engagement, Focusing, and Evoking on the First Target Behavior (Sexual Health or Substance Use)

The interventionist will remind the participant about the YMHP structure and limits of confidentiality. The interventionist will engage in a priorities card sorting activity to identify participant’s 3 main values, which will guide future motivational enhancement and begin to elicit the participant’s view of their first target behavior. After identifying whether they will first tackle sexual health or substance use, the interventionist will assess the participant’s readiness for change, discuss a plan for moving forward, and consider goals. At the end of the session, the interventionist will offer a closing summary and schedule the next session for the following week.

##### Session 2: Second Target Behavior; Continue Focusing and Evoking

The second session will begin with an opening statement to highlight any change talk [50] or goal that was mentioned in the previous session. This session will center primarily on introducing the second target behavior and collaborating on setting the session agenda with the participant. The interventionist will briefly revisit the previous session’s topic and check for any changes or recent events since they last met. Next, they will ask permission to address the second target behavior, all the while using strategies for eliciting change talk and managing discord. Finally, the interventionist will offer a closing summary at the end of the session, highlighting change talk and any steps taken toward change, and schedule the third session during month 1.

##### Session 3: Change Plan

This session will begin by highlighting and integrating change talk on both target behaviors and any ideas or goals the participant has articulated around these behaviors. This will be done collaboratively to set the agenda for the session together. The interventionist will ask permission to discuss collaborating on a change plan together based on the participant’s progress over the last 2 weeks on both goals. Through evoking the participant’s ideas about change, the interventionist will reinforce their self-efficacy for taking steps toward change and offer suggestions on how to adhere to their plan. The interventionist will elicit feedback on a change plan that reflects the participant’s goals, priorities, and readiness to change while anticipating and planning for barriers that may arise in the future. At the end of the session, the interventionist will offer a closing summary highlighting change talk, briefly reiterating the basic plan, and reminding them that the next session will be focused on moving ahead independently.

##### Session 4: Consolidating Commitment; Relapse Prevention or Termination

The final YMHP session will open with the interventionist summarizing the plan developed previously, collaborating on the session agenda based on the participant’s goal updates, and reiterating that this will be the last YMHP session. The participant will discuss how the plan has gone, and the interventionist will manage discord as appropriate while affirming the participant for any steps moving forward. The interventionist will evoke the participant’s ideas about how to adapt the plan in the future should problems arise and develop a backup plan as appropriate. The session will end with a progress summary of the last 4 sessions and a space to reflect on the process of termination with the participant.

### Automated SMS Text Messages (uTECH+YMHP Participants)

Participants assigned to the uTECH+YMHP intervention will receive approximately 2 SMS text messages per week that provide resources promoting sexual wellness and substance use harm reduction. Participants are assigned to specific subgroups based on their answers to the baseline interview to ensure that SMS text messages are tailored. Participants are compensated US $1 per SMS text message to which they respond, and this amount is disbursed at 4 time points corresponding to their 3-, 6-, 9-, and 12-month assessments.

### Incentives

Incentives vary according to intervention and phone type (Figure 1). Participants assigned to the YMHP-only condition will receive US $350, and participants assigned to the uTECH+YMHP condition will receive US $450, including responses to all uTECH messages. The payments for baseline (US $30), 6-month (US $40), and 12-month (Android: US $50; iOS: US $55) interviews are escalated to promote retention in the study. Participants will be paid for each study activity to encourage engagement.

### Discharge From the Study

Participants will be discharged from the study after they complete their 12-month assessment and offboarding. At their final interview, Android-using participants will receive instructions on how to uninstall the eWellness app from their phone. The study team will also discontinue sending automated SMS text messages to uTECH+YMHP participants.

### Ethical Considerations

The research and ethics presented in this study were reviewed and approved by the South Campus institutional review board of the UCLA (#22-000009). The research has also been granted a Certificate of Confidentiality from the National Institutes of Health (CC-OD-22-3555), which deems identifiable information gathered in the course of research not admissible as evidence in any legal processes. Additional terms were provided to ensure confidentiality for the participants. These terms included protection for all study-related data obtained by all uTECH study staff, which includes employees, students, interventionists, and other professionals staffing the project. All participant data will be deidentified and accessible only to staff who completed training in Health Insurance Portability and Accountability Act guidelines and human participants research ethics.

Technical concerns related to data privacy were considered throughout the app development process, which occurred during phase 1 of this project. In collaboration with the UCLA Information Technology and UCLA Compliance departments, considerations related to data storage and data in transit were considered, including the institution’s legal and ethical duties related to the handling of personally identifiable information. Procedures and protocols were established in collaboration with the UCLA Technology Development Group and the UCLA Departments of Computer Science and Engineering.

To enroll in the study, participants would have to review and consent to a study consent form detailing all activities associated with study participation; potential risks and benefits to participation; incentives; and privacy or confidentiality protections granted by the National Institutes of Health Certificate of Confidentiality, UCLA Office of Human Research Protection Program, and study investigators. Participants will be informed during the consenting process that all payments will be made via e-gift card disbursement with a breakdown of all payments totaling the amount of US $350 or US $450 depending on the study group assignment and phone type.

### Primary Outcomes

The primary outcomes that the study will measure are the acceptability, appropriateness, and feasibility of the implementation for uTECH+YMHP and YMHP-only conditions. These primary outcomes will be assessed through surveys conducted at 3-, 6-, 9-, and 12-month time points. The study will measure how outcomes differ between uTECH+YMHP and YMHP-only conditions.

### Secondary Outcomes

The overall framework of this study recognizes that substance use and sexual risk behaviors are primary drivers of HIV and other STIs, which will be assessed through self-reported data on sexual risk, substance use, and self-reported HIV and STI diagnosis, verified through independent medical chart review when submitted by participants. Intimate partner violence, stigma, sociodemographic factors, and use of web-based venues for sex or drug seeking are potential risk factors for HIV and other STIs [63-66]. PrEP use, vaccination, and condom use are potential health protective behaviors. For all participants, we posit that YMHP can also help participants make positive changes toward their self-defined substance use and sexual health goals. For participants in the uTECH+YMHP condition, uTECH can improve participants’ skills and motivation to make behavioral changes to improve their health, which may lessen their risk of acquiring HIV and other STIs. In addition, we posit that in this combined condition, positive behavior changes may be more sustainable and durable compared to receiving YMHP without uTECH.

### Assessment Measures (Baseline and 3-, 6-, 9-, and 12-Month Time Points)

This study will include 3 types of assessment: interviews, web-based surveys, and HIV risk assessments. Interviews (approximately 30 min to 1 h) will be completed at baseline and at 6-month and 12-month time points. Self-completed web-based surveys (approximately 15-30 min) will be completed at 3-month and 9-month time points. HIV risk assessment surveys (approximately 5 min) will be completed at all 5 time points (Table 2).

**Table 2:** Primary and secondary outcomes of the study.

Outcomes			Measures		Variables
**Primary**					
	Acceptability, appropriateness, and feasibility of uTECH SMS text messages	Adapted from AIM^a^, IAM^b^, and FIM^c^ [65]		5-item Likert scale	
	Acceptability, appropriateness, and feasibility of Young Men’s Health Project	Adapted from AIM, IAM, and FIM [65]		5-item Likert scale	
**Secondary**					
	Sociodemographic	Multiple items		Nominal	
	Sexual risk behavior	Multiple items		Nominal, continuous, and dichotomous	
	Intimate partner violence	Intimate partner violence among gay and bisexual men scale [67]		Ordinal	
	PrEP^d^ and PEP^e^ use	Self-reported PrEP or PEP use history		Nominal, continuous; dichotomous, and 6-item Likert scale	
	STDs^f^	Self-reported STD diagnosis history		Nominal, integer, dichotomous, and interval	
	PrEP stigma	PrEP Stigma Scale [68]		5-item Likert scale	
	Substance use	The Alcohol, Smoking, and Substance Involvement Screening Test [69]		Nominal, dichotomous, and yes or no for specific substances	
	COVID-19	Self-reported COVID-19 diagnosis; how COVID-19 has impacted sexual behavior		Dichotomous and qualitative	
	Mpox (monkeypox)	Vaccination status, history of mpox diagnosis and exposure, perceived risk, and stigma		Dichotomous, nominal, interval, and 5-item Likert scale	
	Vaccine confidence	Vaccine Confidence Index [70]		5-item Likert scale	
	Vaccination history	Self-reported vaccination status for hepatitis A, hepatitis B, human papillomavirus, meningococcal B, meningococcal A, C, W, or Y, and COVID-19		Dichotomous and yes or no for specific vaccines	
	Seeking sex or drugs on social networks	Websites, chat rooms, message boards, and apps used to meet partners for sex or partying		Nominal	
	eWellness app features	Types of features (ie, notifications and device functions) and frequency or time of day of notifications that participant would be comfortable with		Nominal, 6-item Likert scale, and dichotomous	
	HIV risk assessment	Men who have sex with men risk index [71] and people who inject drug (injection drug user) risk index [72]		Nominal and ordinal	

^a^AIM: Acceptability of Intervention Measure.

^b^IAM: Intervention Appropriateness Measure.

^c^FIM: Feasibility of Intervention Measure.

^d^PrEP: pre-exposure prophylaxis.

^e^PEP: postexposure prophylaxis.

^f^STD: sexually transmitted disease.

### Expected Timeline

Development of the initial version of eWellness was completed in October 2020, and enrollment for the trial started soon after. The eWellness app continued to undergo refinement and development until phase 2 began in November 2021. Enrollment will continue until the target sample size is reached, with 12 months of study engagement per participant. We expect to complete data collection 12 months after the last participant has been enrolled in the study, that is, by June 2024, and plan the dissemination of results subsequently.

### Statistical Analyses

Statistical methods, models, and procedures will be selected according to the research hypotheses being tested and the types of measures involved. We will set α<.05 as the level for statistical significance but will use appropriate corrections for multiple comparisons (eg, Bonferroni and Hochberg correction [73]) to ensure that the actual overall effective type 1 error remains at an α=.05 level for the tests to be conducted. The frequency and patterns of missing data will be carefully evaluated. To avoid potential bias resulting from missing data, imputation (eg, hot deck or multiple imputation [74]) will be conducted, and statistical significance tests and modeling will be conducted with and without the imputed values. The findings from these analyses will be compared, and any differences will be evaluated. Potential nonlinear relationships between continuous predictors (eg, participants’ age) and outcomes (eg, acceptability, appropriateness, and feasibility of uTECH and YMHP) will be evaluated through spline functions (eg, cubic spline).

### Planned Analytic Approach

Primary outcomes for the study include the acceptability, appropriateness, and feasibility of each intervention condition. In addition, we will examine behavioral health outcomes (sexual risk and protective behaviors and substance use) in both intervention conditions. Tests of fixed effects for estimates of time-specific differences will be performed using appropriate contrasts. Trend changes and their significance will be analyzed, driven by variance components (random effects). We will use growth curve modeling [75] to examine the effects of treatment on outcomes. Growth curve factors also account for the covariation of follow-up observations within participants. Data from 3-, 6-, 9-, and 12-month follow-up assessments will be modeled using latent growth factors using R statistical software [76] (version 4.4.0; R Foundation for Statistical Computing). Because the first nonbaseline time point in the latent growth curve will be the 3-month follow-up, significant differences in the intercept factor would indicate significant postintervention effects. Significant differences in slope factors would indicate that the groups follow different trajectories. When this is noted, potential group differences at each follow-up assessment will be evaluated by varying the location of the referent time point.

### Missing Data

We will impute any missing values, based on the characteristics of the data and the assumptions of the final analyses, such as using multiple imputation if data are missing at random [77]. While analyses of treatment effects are questions of differences after the intervention, baseline data will be used to inform and supplement analyses. For example, baseline data will be used to evaluate whether randomization successfully equated groups on demographic variables and other covariates of theoretical significance. We will use baseline data to evaluate correlates of differential attrition, if observed. If randomization fails or attrition produces group differences, models will use baseline data to control for covariates that are nonorthogonal with treatment condition. We will use growth mixture modeling techniques, including latent class growth curve modeling, to evaluate any meaningful patterns in outcome variable responses across time. We will examine whether treatment condition is associated with outcome response pattern.

### Mediation and Moderation

For outcomes where statistically significant differences in acceptability, appropriateness, and feasibility are observed (ie, significant regression coefficients for the treatment variable associated with the participant-level intercept or slope growth factors), post hoc analysis will examine the roles of substance use and technology use as potential mediators. A primary assumption is that uTECH will be associated with improvement in awareness of how technology influences engagement in HIV risk and substance use behavior [78-80]. For each mediator, validated substance use and technology reports will be entered into the model. Like the approach described for primary outcomes, we will specify latent growth curve factors for the hypothesized mediators to understand the covariation of observations within individuals across time. Subsequently, we will examine whether initial differences in the mediators or changes in the mediators over time account for associations between the outcome variable and the treatment conditions. We will use strategies for assessing time-varying mediators that permit regression coefficients of the outcome on the mediator to vary over time. Where appropriate, we will examine the utility of applying these techniques. We will use parameter constraint approaches [81] to assess the statistical significance of indirect pathways. Bootstrapping [82] tests of mediation cannot be conducted with latent variable mediators and nonnormal exogenous variables. When appropriate, we will use alternative approaches such as Bayesian structural equation modeling and Monte Carlo simulation methods in the presence of nonnormality and complex latent variable structures [83]. Participant-level reports of substance use and technology use will be entered into the models, and we will examine whether initial levels of substance use and technology use (or changes in these variables over time) moderate the association between primary outcome variables and treatment condition by examining the significance of interaction terms between growth factors.

### Sample Size Calculation and Power Analysis

For the power calculation, we used 1 primary outcome, acceptability. We used a balanced repeated measures design [84] with pretreatment baseline measurements. Using a logistic regression generalized linear mixed model [85], we allowed an individual’s 5 measurements (baseline and 3-, 6-, 9-, and 12-month) for acceptability to be correlated via an autoregressive-1 structure. With an initial sample size of 330 (to account for 95% retention), an autocorrelation of 0.1, and a baseline effectiveness of 85%, we aimed to detect a minimum time-averaged difference of 9% in acceptability between any 2 conditions, maintaining 80% power and an a priori α level of .05. This calculation was based on pairwise comparisons between uTECH+YMHP versus YMHP-only conditions. If the autocorrelation [86] between measures within the same individual was as high as 0.5, the sample size was adjusted to detect a minimum difference of 11% between the 2 conditions. A 9% to 11% difference between the 2 conditions is considered statistically significant, given the existing evidence supporting YMHP and the relative cost-effectiveness and ease of delivery of the uTECH intervention. The ability to demonstrate a small increase in the acceptability of uTECH over YMHP will support the widespread implementation of uTECH in future efficacy trials.

## Results

This study was funded in August 2019. Between August 2019 and June 2024, we have enrolled 424 participants on a rolling basis. Approximately 71% (301/424) of the study participants have completed 12 months of participation. Data collection concluded in June 2024. Data analysis is currently under way, and expected results will be published in the fall of 2025.

## Discussion

### Anticipated Findings

As mentioned in the *Results* section, at the time of writing this manuscript, the study was fully enrolled (N=424), which meets and exceeds the enrollment objective of 330 participants to account for attrition. Data analysis activities to evaluate the stated study hypotheses are currently underway.

uTECH is a response to changing internet or social media habits and aims to leverage ML-enhanced technology for personalized and automated HIV prevention and substance use harm reduction. Websites and social media use recommendation algorithms to personalize nearly every aspect of our web-based lives, from what news we see to what products we want to buy. All internet users already interact with websites and social media that use ML algorithms to predict and personalize content for their users based on their individual preferences, purchases, and habits. To do this, ML requires an enormous amount of personal data to make accurate and meaningful predictions. The widespread use of recommendation algorithms in social media and websites has also changed how people use the internet, and algorithm-mediated personalized content is not only widely accepted but is also increasingly preferred by people, especially young adults [87], for consuming media and content. According to a consumer research survey, the vast majority of users are willing to share personal data in exchange for better, more relevant, and curated digital experiences [88].

There are potential challenges to the success of uTECH’s implementation trial. First, it uses convenience sampling techniques to recruit the target population, which limits the generalizability of the results. In addition, even if recruitment efforts enable us to successfully meet our planned enrollment target, participant data could still fall short of what is needed for ML or for adequate statistical power to detect the effects of intervention conditions on outcomes. For example, numerous technical issues could impact data collection. The eWellness app requires additional battery power as it runs in the background of mobile phones. Certain phone models may automatically turn off data collection to optimize battery use. Participants could also turn off the eWellness app themselves and forget or be unable to turn it back on.

For iOS users, we rely on the availability of third-party data, which may sometimes be unavailable. For example, during leadership changes at Twitter, mass system outages occurred, which impacted the ability of some participants to download their data. Some data downloads (eg, from Grindr) take an unknown amount of time to become available to access and are sometimes a greater burden for participants because it requires the participant to log in to access and download their data and then send them to our secure, encrypted email. This process takes some time, and the access could expire if participants miss their email notification from the third-party system. Despite incentives, many participants may not complete all data collection activities, resulting in missing data. Among these, missing SMS text message feedback may impact adaptive ML and thus reduce the personalization and relevance of the SMS text messages participants receive.

This research will contribute as an implementation science demonstration project on the acceptability, appropriateness, and feasibility of an intervention that delivers a moderate to high level of relevance and personalization that requires fewer direct contact hours and has the potential to reach a far greater number of participants than structured, multisession interventions or in-person interventions. In a less-resourced environment or as public funding becomes more constrained, high-impact interventions that require less staffing may be a more efficient option compared to traditional HIV and substance use intervention paradigms. In addition, this work can potentially be adapted for other public health promotion efforts, including chronic disease management, HIV care adherence, and vaccination promotion.

### Conclusions

The primary objective of this study is to evaluate the acceptability, appropriateness, and feasibility of uTECH, a text message–based risk reduction intervention that leverages an ML and data mining algorithm to provide personalized prevention messaging to SGM participants. To our knowledge, this is the first study to use ML and digital technologies to provide tailored sexual and substance use harm reduction for these populations. The results of this implementation trial will offer insights for future research within this field, contributing to developments that can reduce HIV risk and substance use. Outcomes and summaries of this implementation trial will be presented, and recommendations for future research will be suggested if any knowledge gaps are identified. The potential findings of this implementation trial will be reported in academic papers, which will be submitted to peer-reviewed journals and presented at national and international conferences.

## Appendix

Multimedia Appendix 1 SPIRIT (Standard Protocol Items: Recommendations for Interventional Trials) checklist.

Multimedia Appendix 2 Peer-review report from the National Institute on Drug Abuse Special Emphasis Panel - National Institute on Drug Abuse (NIDA).

## Abbreviations

CDC: Centers for Disease Control and Prevention
EBRRI: evidence-based risk reduction intervention
IMB: Information-Motivation-Behavior Skills
MI: motivational interviewing
ML: machine learning
MSM: men who have sex with men
NIH: National Institutes of Health
PrEP: pre-exposure prophylaxis
SGM: sexual and gender minority
SPIRIT: Standard Protocol Items: Recommendations for Interventional Trials
STI: sexually transmitted infection
UCLA: University of California, Los Angeles
YMHP: Young Men’s Health Project

## Data Availability

The data sets generated during and analyzed during this study are not publicly available due to the sensitive nature of the data but are available from the corresponding author on reasonable request.

## References

[1] Hess KL, Hu X, Lansky A, Mermin J, Hall HI (2017). Lifetime risk of a diagnosis of HIV infection in the United States. Ann Epidemiol.

[2] (2024). HIV surveillance report: diagnoses, deaths, and prevalence of HIV in the United States and 6 territories and freely associated states, 2022. Centers for Disease Control and Prevention.

[3] Herbst JH, Jacobs ED, Finlayson TJ, McKleroy VS, Neumann MS, Crepaz N, HIV/AIDS Team (2008). Estimating HIV prevalence and risk behaviors of transgender persons in the United States: a systematic review. AIDS Behav.

[4] HIV infection, risk, prevention, and testing behaviors among transgender women: national HIV behavioral surveillance 7 U.S. cities, 2019–20. Centers for Disease Control and Prevention.

[5] Rivera AV, Harriman G, Carrillo SA, Braunstein SL (2021). Trends in methamphetamine use among men who have sex with men in New York City, 2004-2017. AIDS Behav.

[6] Strathdee SA, Bristow CC, Gaines T, Shoptaw S (2021). Collateral damage: a narrative review on epidemics of substance use disorders and their relationships to sexually transmitted infections in the United States. Sex Transm Dis.

[7] Luk JW, Worley MJ, Winiger E, Trim RS, Hopfer CJ, Hewitt JK, Brown SA, Wall TL (2016). Risky driving and sexual behaviors as developmental outcomes of co-occurring substance use and antisocial behavior. Drug Alcohol Depend.

[8] Halkitis PN, Mukherjee PP, Palamar JJ (2009). Longitudinal modeling of methamphetamine use and sexual risk behaviors in gay and bisexual men. AIDS Behav.

[9] Colfax G, Shoptaw S (2005). The methamphetamine epidemic: implications for HIV prevention and treatment. Curr HIV/AIDS Rep.

[10] Chaudhry AB, Reisner SL (2019). Disparities by sexual orientation persist for major depressive episode and substance abuse or dependence: findings from a national probability study of adults in the United States. LGBT Health.

[11] Kerridge BT, Pickering RP, Saha TD, Ruan WJ, Chou SP, Zhang H, Jung J, Hasin DS (2017). Prevalence, sociodemographic correlates and DSM-5 substance use disorders and other psychiatric disorders among sexual minorities in the United States. Drug Alcohol Depend.

[12] Woody GE, VanEtten-Lee ML, McKirnan D, Donnell D, Metzger D, Seage G, Gross M (2001). Substance use among men who have sex with men: comparison with a national household survey. J Acquir Immune Defic Syndr.

[13] Brennan J, Kuhns LM, Johnson AK, Belzer M, Wilson EC, Garofalo R (2012). Syndemic theory and HIV-related risk among young transgender women: the role of multiple, co-occurring health problems and social marginalization. Am J Public Health.

[14] American Psychiatric Association (2013). Diagnostic And Statistical Manual Of Mental Disorders (DSM-5). 5th edition.

[15] Richards C, Bouman WP, Seal L, Barker MJ, Nieder TO, T'Sjoen G (2016). Non-binary or genderqueer genders. Int Rev Psychiatry.

[16] Brown A Lesbian, gay and bisexual online daters report positive experiences – but also harassment. Pew Research Center.

[17] Murphy M, Tao J, Goedell WC, Berk J, Chu CT, Nunn A, Sosnowy C, Chan P (2021). Characterizing substance use among men who have sex with men presenting to a sexually transmitted infection clinic. Int J STD AIDS.

[18] Zou H, Fan S (2017). Characteristics of men who have sex with men who use smartphone geosocial networking applications and implications for HIV interventions: a systematic review and meta-analysis. Arch Sex Behav.

[19] Beymer MR, Weiss RE, Bolan RK, Rudy ET, Bourque LB, Rodriguez JP, Morisky DE (2014). Sex on demand: geosocial networking phone apps and risk of sexually transmitted infections among a cross-sectional sample of men who have sex with men in Los Angeles County. Sex Transm Infect.

[20] DeVost MA, Beymer MR, Weiss RE, Shover CL, Bolan RK (2018). App-based sexual partner seeking and sexually transmitted infection outcomes: a cross-sectional study of HIV-negative men who have sex with men attending a sexually transmitted infection clinic in Los Angeles, California. Sex Transm Dis.

[21] Jalil EM, Torres TS, de A Pereira CC, Farias A, Brito JD, Lacerda M, da Silva DA, Wallys N, Ribeiro G, Gomes J, Odara T, Santiago L, Nouveau S, Benedetti M, Pimenta C, Hoagland B, Grinsztejn B, Veloso VG (2022). High rates of sexualized drug use or chemsex among Brazilian transgender women and young sexual and gender minorities. Int J Environ Res Public Health.

[22] Maxwell S, Shahmanesh M, Gafos M (2019). Chemsex behaviours among men who have sex with men: a systematic review of the literature. Int J Drug Policy.

[23] Correia RB, Wood IB, Bollen J, Rocha LM (2020). Mining social media data for biomedical signals and health-related behavior. Annu Rev Biomed Data Sci.

[24] Correia RB, Li L, Rocha LM (2016). Monitoring potential drug interactions and reactions via network analysis of Instagram user timelines. Pac Symp Biocomput.

[25] Weber I, Achananuparp P (2016). Insights from machine-learned diet success prediction. Pac Symp Biocomput.

[26] Li J, Xu Q, Cuomo R, Purushothaman V, Mackey T (2020). Data mining and content analysis of the Chinese social media platform Weibo during the early COVID-19 outbreak: retrospective observational infoveillance study. JMIR Public Health Surveill.

[27] Velasco E, Agheneza T, Denecke K, Kirchner G, Eckmanns T (2014). Social media and internet-based data in global systems for public health surveillance: a systematic review. Milbank Q.

[28] Maloney KM, Bratcher A, Wilkerson R, Sullivan PS (2020). Electronic and other new media technology interventions for HIV care and prevention: a systematic review. J Int AIDS Soc.

[29] Meiksin R, Melendez-Torres GJ, Falconer J, Witzel TC, Weatherburn P, Bonell C (2021). eHealth interventions to address sexual health, substance use, and mental health among men who have sex with men: systematic review and synthesis of process evaluations. J Med Internet Res.

[30] Mitchell TM (1997). Machine Learning.

[31] Włodarczyk T, Płotka S, Szczepański T, Rokita P, Sochacki-Wójcicka N, Wójcicki J, Lipa M, Trzciński T (2021). Machine learning methods for preterm birth prediction: a review. Electronics.

[32] Ali F, El-Sappagh S, Islam SR, Ali A, Attique M, Imran M, Kwak KS (2021). An intelligent healthcare monitoring framework using wearable sensors and social networking data. Future Gener Comput Syst.

[33] Lopez-Castroman J, Moulahi B, Azé J, Bringay S, Deninotti J, Guillaume S, Baca-Garcia E (2020). Mining social networks to improve suicide prevention: a scoping review. J Neurosci Res.

[34] Ovalle A, Goldstein O, Kachuee M, Wu ES, Hong C, Holloway IW, Sarrafzadeh M (2021). Leveraging social media activity and machine learning for HIV and substance abuse risk assessment: development and validation study. J Med Internet Res.

[35] Garofalo R, Kuhns LM, Reisner SL, Biello K, Mimiaga MJ (2018). Efficacy of an empowerment-based, group-delivered HIV prevention intervention for young transgender women: the project LifeSkills randomized clinical trial. JAMA Pediatr.

[36] Parsons JT (2015). Young men's health project. Centers for Disease Control and Prevention.

[37] Parsons JT, Starks T, Gurung S, Cain D, Marmo J, Naar S (2019). Clinic-based delivery of the young men's health project (YMHP) targeting HIV risk reduction and substance use among young men who have sex with men: protocol for a type 2, hybrid implementation-effectiveness trial. JMIR Res Protoc.

[38] Outlaw AY, Naar-King S, Parsons JT, Green-Jones M, Janisse H, Secord E (2010). Using motivational interviewing in HIV field outreach with young African American men who have sex with men: a randomized clinical trial. Am J Public Health.

[39] Alemagno SA, Stephens RC, Stephens P, Shaffer-King P, White P (2009). Brief motivational intervention to reduce HIV risk and to increase HIV testing among offenders under community supervision. J Correct Health Care.

[40] Stephenson R, Todd K, Kahle E, Sullivan SP, Miller-Perusse M, Sharma A, Horvath KJ (2020). Project moxie: results of a feasibility study of a telehealth intervention to increase HIV testing among binary and nonbinary transgender youth. AIDS Behav.

[41] Taylor RD, Bimbi DS, Joseph HA, Margolis AD, Parsons JT (2011). Girlfriends: evaluation of an HIV-risk reduction intervention for adult transgender women. AIDS Educ Prev.

[42] Chan AW, Tetzlaff JM, Altman DG G, Laupacis A, Gøtzsche PC, Krleža-Jerić K, Hróbjartsson A, Mann H, Dickersin K, Berlin JA, Doré CJ, Parulekar WR, Summerskill WSM, Groves T, Schulz KF, Sox HC, Rockhold FW, Rennie D, Moher D (2013). SPIRIT 2013 statement: defining standard protocol items for clinical trials. Ann Intern Med.

[43] What is AWARE?. AWARE: Open-source Context Instrumentation Framework For Everyone.

[44] Moshe I, Terhorst Y, Opoku Asare K, Sander LB, Ferreira D, Baumeister H, Mohr DC, Pulkki-Råback L (2021). Predicting symptoms of depression and anxiety using smartphone and wearable data. Front Psychiatry.

[45] Opoku Asare K, Terhorst Y, Vega J, Peltonen E, Lagerspetz E, Ferreira D (2021). Predicting depression from smartphone behavioral markers using machine learning methods, hyperparameter optimization, and feature importance analysis: exploratory study. JMIR Mhealth Uhealth.

[46] Vega J (2016). Monitoring Parkinson's disease progression using behavioural inferences, mobile devices and web technologies. Proceedings of the 25th International Conference Companion on World Wide Web.

[47] Bae S, Chung T, Ferreira D, Dey AK, Suffoletto B (2018). Mobile phone sensors and supervised machine learning to identify alcohol use events in young adults: implications for just-in-time adaptive interventions. Addict Behav.

[48] Hamilton AB, Finley EP (2019). Qualitative methods in implementation research: an introduction. Psychiatry Res.

[49] Rubak S, Sandbaek A, Lauritzen T, Christensen B (2005). Motivational interviewing: a systematic review and meta-analysis. Br J Gen Pract.

[50] Miller WR, Rollnick S (2013). Motivational Interviewing: Helping People Change. 3rd edition.

[51] Fisher WA, Fisher JD, Harman J, Suls J, Wallston KA (2003). The information-motivation-behavioral skills model: a general social psychological approach to understanding and promoting health behavior. Social Psychological Foundations of Health and Illness.

[52] Cornman DH, Kiene SM, Christie S, Fisher WA, Shuper PA, Pillay S, Friedland GH, Thomas CM, Lodge L, Fisher JD (2008). Clinic-based intervention reduces unprotected sexual behavior among HIV-infected patients in KwaZulu-Natal, South Africa: results of a pilot study. J Acquir Immune Defic Syndr.

[53] Cosio D, Heckman TG, Anderson T, Heckman BD, Garske J, McCarthy J (2010). Telephone-administered motivational interviewing to reduce risky sexual behavior in HIV-infected rural persons: a pilot randomized clinical trial. Sex Transm Dis.

[54] Pearson CR, Micek MA, Simoni JM, Hoff PD, Matediana E, Martin DP, Gloyd SS (2007). Randomized control trial of peer-delivered, modified directly observed therapy for HAART in Mozambique. J Acquir Immune Defic Syndr.

[55] Margolin A, Avants SK, Warburton LA, Hawkins KA, Shi J (2003). A randomized clinical trial of a manual-guided risk reduction intervention for HIV-positive injection drug users. Health Psychol.

[56] Parsons JT, Golub SA, Rosof E, Holder C (2007). Motivational interviewing and cognitive-behavioral intervention to improve HIV medication adherence among hazardous drinkers: a randomized controlled trial. J Acquir Immune Defic Syndr.

[57] Purcell DW, Latka MH, Metsch LR, Latkin CA, Gómez CA, Mizuno Y, Arnsten JH, Wilkinson JD, Knight KR, Knowlton AR, Santibanez S, Tobin KE, Rose CD, Valverde EE, Gourevitch M, Eldred L, Borkowf CB (2007). Results from a randomized controlled trial of a peer-mentoring intervention to reduce HIV transmission and increase access to care and adherence to HIV medications among HIV-seropositive injection drug users. J Acquir Immune Defic Syndr.

[58] The next generation of business text messaging services. Twilio.

[59] Make every interaction an experience that matters. Qualtrics.

[60] Our products help businesses detect fraud, safeguard users, and protect revenues. Scamalytics.

[61] Easy scheduling ahead. Calendly.

[62] (2021). Research participant bill of rights/experimental subjects bill of rights. UCLA Research Administration Human Research Protection Program.

[63] Beadnell B, Baker SA, Morrison DM, Knox K (2000). HIV/STD risk factors for women with violent male partners. Sex Role.

[64] Cunningham SD, Kerrigan DL, Jennings JM, Ellen JM (2009). Relationships between perceived STD-related stigma, STD-related shame and STD screening among a household sample of adolescents. Perspect Sex Reprod Health.

[65] Weiner BJ, Lewis CC, Stanick C, Powell BJ, Dorsey CN, Clary AS, Boynton MH, Halko H (2017). Psychometric assessment of three newly developed implementation outcome measures. Implement Sci.

[66] Wang H, Zhang L, Zhou Y, Wang K, Zhang X, Wu J, Wang G (2018). The use of geosocial networking smartphone applications and the risk of sexually transmitted infections among men who have sex with men: a systematic review and meta-analysis. BMC Public Health.

[67] Stephenson R, Finneran C (2013). The IPV-GBM scale: a new scale to measure intimate partner violence among gay and bisexual men. PLoS One.

[68] Siegler AJ, Wiatrek S, Mouhanna F, Amico KR, Dominguez K, Jones J, Patel RR, Mena LA, Mayer KH (2020). Validation of the HIV pre-exposure prophylaxis stigma scale: performance of Likert and semantic differential scale versions. AIDS Behav.

[69] Humeniuk R, Henry-Edwards S, Ali R, Poznyak V, Monteiro MG (2010). The alcohol, smoking and substance involvement screening test (ASSIST): manual for use in primary care. World Health Organization.

[70] Frew PM, Holloway IW, Goldbeck C, Tan D, Wu E, Jauregui J, Fenimore VL, Randall LA, Lutz CS, Mendel J, Aikin AL, Nowak GJ, Bednarczyk RA (2018). Development of a measure to assess vaccine confidence among men who have sex with men. Expert Rev Vaccines.

[71] Smith DK, Pals SL, Herbst JH, Shinde S, Carey JW (2012). Development of a clinical screening index predictive of incident HIV infection among men who have sex with men in the United States. J Acquir Immune Defic Syndr.

[72] Smith DK, Pan Y, Rose CE, Pals SL, Mehta SH, Kirk GD, Herbst JH (2015). A brief screening tool to assess the risk of contracting HIV infection among active injection drug users. J Addict Med.

[73] Haynes W, Dubitzky W, Wolkenhauer O, Cho KH, Yokota H (2013). Benjamini–Hochberg method. Encyclopedia of Systems Biology.

[74] Kang H (2013). The prevention and handling of the missing data. Korean J Anesthesiol.

[75] Ram N, Grimm KJ (2009). Growth mixture modeling: a method for identifying differences in longitudinal change among unobserved groups. Int J Behav Dev.

[76] R Core Team The R project for statistical computing. Cran R.

[77] Azur MJ, Stuart EA, Frangakis C, Leaf PJ (2011). Multiple imputation by chained equations: what is it and how does it work?. Int J Methods Psychiatr Res.

[78] Castillo AG, Jandorf L, Thélémaque LD, King S, Duhamel K (2012). Reported benefits of participation in a research study. J Community Health.

[79] Cornelius JB, Smoot JM (2022). The impact of technology on adolescent sexual and reproductive needs. Int J Environ Res Public Health.

[80] Moor L, Anderson JR, Power J, James A, Waling A, Shackleton N (2023). The risks and benefits of technologised sexual practice scale: a quantitative measure of technology facilitated sex and intimacy. Sex Health.

[81] Niculescu RS, Mitchell TM, Rao RB (2006). Bayesian network learning with parameter constraints. J Mach Learn Res.

[82] Wood RE, Goodman JS, Beckmann N, Cook A (2007). Mediation testing in management research. Organ Res Methods.

[83] Palomo J, Dunson DB, Bollen K, Sik-Yum L (2007). Bayesian structural equation modeling. Handbook of Latent Variable and Related Models.

[84] Afsarinejad K (1983). Balanced repeated measurements designs. Biometrika.

[85] Koosha M, Amiri A (2012). Generalized linear mixed model for monitoring autocorrelated logistic regression profiles. Int J Adv Manuf Technol.

[86] Sideridis GD, Greenwood CR (1997). Is human behavior autocorrelated? an empirical analysis. J Behav Educ.

[87] Perez Vallejos E, Dowthwaite L, Creswich H, Portillo V, Koene A, Jirotka M, McCarthy A, McAuley D (2021). The impact of algorithmic decision-making processes on young people's well-being. Health Informatics J.

[88] Zoghby J, Tieman S, Moiño JP (2018). Pulse check. Accenture Interactive.

