# Supplementary material for: Novel Machine Learning HIV Intervention for Sexual and Gender Minority Young People Who Have Sex With Men (uTECH): Protocol for a Randomized Comparison Trial
Source: JMIR Res Protoc. 2024 Aug 20;13:e58448. doi: 10.2196/58448 (PMC11372318; doi:10.2196/58448)
Supplement: Multimedia Appendix 2 [file resprot_v13i1e58448_app2.pdf]

SUMMARY STATEMENT

(Privileged Communication)

PROGRAM CONTACT:
Richard Jenkins
301-443-6504
jenkinsri@mail.nih.gov

Release Date: 03/15/2019
Revised Date:

Application Number: 1 DP2 DA049296-01

Principal Investigator

HOLLOWAY, IAN WALTER

Applicant Organization: UNIVERSITY OF CALIFORNIA LOS ANGELES

Review Group: ZDA1 HXO-H (11)
National Institute on Drug Abuse Special Emphasis Panel
Avenir Award Program for Research on Substance Abuse and HIV/AIDS (DP2) applications
AIDS

Meeting Date: 03/14/2019
Council: MAY 2019
Requested Start: 07/01/2019

RFA/PA: DA18-004
PCC: CV/RJP

Project Title: uTECH: Machine Learning for HIV Prevention Among Substance Using GBMSM

SRG Action: Impact Score:23
Next Steps: Visit https://grants.nih.gov/grants/next_steps.htm
Human Subjects: 30-Human subjects involved - Certified, no SRG concerns
Animal Subjects: 10-No live vertebrate animals involved for competing appl.
Gender: 3A-Only men, scientifically acceptable
Minority: 1A-Minorities and non-minorities, scientifically acceptable
Children: 3A-No children included, scientifically acceptable

Project Year	Direct Costs Requested	Estimated Total Cost
1	1,500,000	1,500,000
TOTAL	1,500,000	1,500,000

ADMINISTRATIVE NOTE: Since the NIH Director's New Innovator Award applications are reviewed differently from other NIH grant mechanisms, criterion scores and percentiles are not assigned. The budget shown above is the requested budget and has not been adjusted to reflect any recommendations made by reviewers. If an award is planned, the costs will be calculated by Institute grants management staff based on the recommendations outlined below in the COMMITTEE BUDGET RECOMMENDATIONS section.
EARLY STAGE INVESTIGATOR
NEW INVESTIGATOR

1DP2DA049296-01 Holloway, Ian

RESUME AND SUMMARY OF DISCUSSION: In this application for a NIDA HIV/AIDS Avenir (DP2) Award, Dr. Holloway from University of California, Los Angeles seeks five years of support to refine and test the acceptability, appropriateness and feasibility of an innovative, social media "big data" machine learning intervention, which aims to reduce HIV transmission risk by integrating biomedical and behavioral risk reduction strategies. With the DP2 interview, the reviewers' assessment of the application improved substantially, in part as a result of Dr. Holloway's clear presentation that answered their questions. The project has significance in that it addresses sexual and drug use risk behaviors for HIV, as well as an important barrier to reaching gay, bisexual and other men who have sex with men (GBMSM) substance users with educational information for HIV prevention and treatment of substance use disorders. It appears to be a large leap forward in the form of a new machine learning technology tailored to the needs of this population, at the same time, is only an incremental step toward an efficacy trial. Dr. Holloway has an outstanding track record, a clear vision, and a unique niche, and has assembled a supportive research team of established collaborators. Innovation is found in: the machine learning/individualized approach to intervention, the implementation approach, and the evaluation of the acceptability, appropriateness, and feasibility of novel interventions as compared to existing interventions. Strengths in the approach include the well described procedures, plans, and analyses, and the user-centered design to refine the uTech intervention, at the same time, privacy issues as well as the unclear rate of uptake and the management of stigmatization are weaknesses. The environment is excellent. Overall, this is an outstanding to excellent application.

DESCRIPTION (provided by applicant): Gay, bisexual and other men who have sex with men (GBMSM) are disproportionately impacted by HIV in the U.S. Substance use is an important influence on HIV risk among GBMSM; and partner seeking for both sex and substance use have largely moved online and to geosocial networking platforms designed for GBMSM (e.g., Grindr). Technological advances in the collection and mining of "big data" to inform behavioral health interventions have increased in recent years but have not been applied directly to HIV prevention and substance use harm reduction among GBMSM. At the same time, despite major advances in biomedical HIV prevention (i.e., pre-exposure prophylaxis [PrEP]) and substance use harm-reduction (i.e., medication assisted therapy [MAT]), these strategies are underutilized by GBMSM. My research team and I conducted formative research on social media data mining and machine learning through a NIDA A/START (R03) to identify patterns of technology use that are associated with HIV risk and substance use among GBMSM. We established computational functionality of a culturally tailored social media data mining program among substance using GBMSM. I now take an important scientific risk to use this technology to develop an HIV prevention intervention for GBMSM, tentatively titled uTECH, that leverages insights from machine learning to trigger personalized intervention content to increase biomedical HIV prevention and substance use harm reduction. Specifically, I propose to conduct a two-phase study. In Phase 1 I will conduct qualitative interviews with GBMSM to inform the iterative development and refinement of uTECH. In Phase 2, I will test the acceptability, appropriateness and feasibility of uTECH in a comparative implementation science trial. For this phase, I will (a) enroll racially diverse, HIV-negative, substance using GBMSM; (b) randomize them to either the uTECH intervention or a comparison group; and (c) measure acceptability, appropriateness and feasibility through 6 months post-intervention. My primary implementation science outcomes will be acceptability (i.e., Acceptability of Intervention Measure [AIM]), appropriateness (i.e., Intervention Appropriateness Measure [IAM]), and feasibility (i.e., Feasibility of Intervention Measure [FIM]). I believe that the power of "big data" and new technologies can be harnessed for effective HIV prevention with substance using GBMSM. In the era of increasing HIV prevention fatigue among GBMSM, the ability to deliver quick, convenient and highly personalized interventions presents an opportunity to reinvigorate HIV prevention.

PUBLIC HEALTH RELEVANCE: Gay, bisexual and other men who have sex with men (GBMSM) are the largest HIV transmission group in the U.S. and substance use is an important factor driving new

HIV infections in this population. The overarching goal of this proposal is to reinvigorate HIV prevention among GBMSM by refining and testing the acceptability, appropriateness and feasibility of an innovative, social media "big data" machine learning intervention, which aims to reduce HIV transmission risk by integrating biomedical and behavioral risk reduction strategies.

CRITIQUE 1

Significance: 2
Investigator(s): 2
Innovation: 5
Approach: 2
Environment: 2

Overall Impact: This is a proposal to develop and test the feasibility of uTECH, a big data intervention for substance-using MSM encouraging uptake of interventions including PrEP and medications for addiction treatment, as well as sexual and drug risk reduction for HIV and overdose prevention. There are two phases to the project: (1) interviews and pilot testing to refine the intervention, (2) a comparative implementation science trial with a motivational enhancement intervention as the control (investigator reports this does not meet clinical trial criteria because outcomes are oriented around acceptability rather than health outcomes; comparator needed to see how acceptability of this compares to existing interventions). The significance of this proposal is based on the need to address HIV risk and substance use among MSM. This has been challenging due in part to intervention fatigue among the targeted population. Reaching people at the places they frequent – online social media services – may be more effective than other ways of reaching them. Furthermore, the intervention itself allows for individualization based on analysis of social media habits and text – this individualization is significant and innovative. The investigators include the PI, who has substantial experience in this area, and an appropriate team of more senior experts. The approach includes refining the intervention and conducting the trial – both are well-described and without any major flaws. The primary concern with this proposal rests on innovation. This appears to be a next step from previous work of the investigator, building on existing efforts much like an R34 would. This seems incremental rather than boldly innovative. In addition, there are some vague concerns regarding rapidly developing issues with privacy in social media, with possible coming changes to user agreements sabotaging this type of work.

1. Significance
Strengths
- Addresses sexual and drug use risk behaviors for HIV, as well as other substance use risks
- Meets target audience where they spend time – selected social media sites
- Individualized intervention

Weaknesses
- Incremental step toward building background data for efficacy trial

2. Investigator(s)
Strengths
- Dr Holloway is a very strong PI for this, with sufficient experience in the fields of social media / big data, and HIV/MSM/substance use
- Team includes Dr Shoptaw, a strong mentor and topical / addiction expert

Weaknesses

- Only included biosketch is for Dr Holloway, thus not possible to evaluate other Co-Is not familiar to this reviewer

3. Innovation
Strengths

- Machine learning/individualized approach to intervention is a strong element of innovation

Weaknesses

- The major weakness to this proposal is that it appears to be an incremental step forward in the research pathway of the principal investigator. Unclear why this would not be considered as an R34, as it appears to be like one. The sample size of >300 also seems to be large for an acceptability/feasibility trial.

4. Approach
Strengths

- Well described details for procedures, plans, and analyses for both phases 1 and 2
- Addresses several concerns that arise, such as the need for a comparator

Weaknesses

- Unclear why not evaluating efficacy outcomes, at least in an exploratory fashion

5. Environment
Strengths

- UCLA has a strong record of work with the target population and a strong collection of appropriate investigators

Weaknesses

- A possible concern would be the changing environment in social media with regards to privacy and use of “big data”

Protections for Human Subjects: Acceptable Risks and/or Adequate Protections

Data and Safety Monitoring Plan (Applicable for Clinical Trials Only): Not Applicable (No Clinical Trials)

Inclusion of Women, Minorities and Children Applicable Only for Human Subjects Research:

G3A - Only Men, Acceptable

M1A - Minority and Non-minority, Acceptable

C3A - No Children Included, Acceptable

Vertebrate Animals

- Not Applicable (No Vertebrate Animals)

Biohazards

- Not Applicable (No Biohazards)

Applications from Foreign Organizations: Not Applicable (No Foreign Organizations)

Resource Sharing Plans:

Unacceptable

- unable to locate

Budget and Period of Support:

- Budget Modifications Recommended (in amount/time)

Recommended budget modifications or possible overlap identified: unable to locate and thus evaluate

Additional Comments to Applicant (Optional):

CRITIQUE 2

Significance: 3
Investigator(s): 2
Innovation: 4
Approach: 4
Environment: 1

Overall Impact: This application for an Avenir Award is submitted by Dr. Ian Holloway, a licensed clinical social worker and a tenured Associate Professor at UCLA. Dr. Holloway has dedicated his career to HIV prevention among MSM, with expertise in social network analysis, and thus is a strong candidate for the Avenir Award. In this proposal, he aims to develop and test a novel social media intervention that uses “big data” machine learning to personalize the intervention content and delivery. This proposal builds upon an ASTART grant, which used mixed methods to examine how social media influences HIV risk among substance using MSM. By mining social media data, his team identified terms and symbols that correlated with HIV risk behaviors. The application of machine learning to social media data is innovative, and his prior work has identified terms and symbols that are associated with risk behaviors. The proposed Avenir project will apply this information to the development of a mobile intervention for at-risk MSM who use social media sites. The PI and his team are well qualified to conduct this project. The public health significance of the proposal is high, as MSM continue to account for most new HIV infections. It is somewhat unclear how much the uTECH intervention has already been developed through the ASTART versus what still needs to be done in the development phase of the proposed project. The overall design of this implementation science trial is robust, but there are several unresolved issues that dampened my enthusiasm for the proposal. This includes limited potential benefits of MAT for a population with very low opioid abuse, a lack of attention to measuring uptake of MAT or PrEP as an outcome of the uTECH intervention, and concerns about user privacy on social media sites and how this will affect broad dissemination. A minor critique is that the PI discusses alternate strategies considered in the development of uTech in his prior study but does not address potential challenges that might arise in the pilot and RCT phases of the Avenir proposal.

1. Significance

Strengths

- MSM continue to be disproportionately affected by the HIV epidemic, accounting for 83% of all new HIV diagnoses in men. Effective prevention interventions to halt HIV transmission in this population are urgently needed. Since social media is popular among MSM, it makes sense to

harness the data available through these sites and to use social media sites as a platform for individualized interventions. If the proposed intervention is effective, the public health impact for MSM would be substantial.

- The proposed intervention attempts to address both substance abuse treatment and HIV prevention. This is highly significant, as substance abuse is common among MSM and drives sexual risk taking.

Weaknesses

- MAT is only helpful for individuals who are dependent on opioids, limiting the applicability of the uTech intervention among MSM.

2. Investigator(s)
Strengths

- The PI is a licensed social worker whose research program has focused on HIV prevention among MSM. He has been productive, including publication of over 50 peer reviewed manuscripts.
- He has a successful track record of funding from the NIH (ASTART), the California HIV/AIDS Research Program, and the UCLA CFAR.
- His ASTART project demonstrates the ability to develop new methods for implementation of technology-delivered interventions for HIV prevention.
- The PI has assembled a strong team with expertise in addiction medicine, intervention development, computer science, machine learning, and implementation science.
- Reference letters are strong, offering confidence in his ability to conduct groundbreaking research.

Weaknesses

- None noted

3. Innovation
Strengths

- The key points of innovation identified by the PI are: (1) integration of multiple biomedical prevention strategies that address both HIV and substance abuse; (2) use of machine learning to mine the “big data” available through social media sites to personalize interventions; and (3) the proposal to implement a comparative acceptability, appropriateness, and feasibility trial.
- The application of machine learning to HIV prevention is indeed novel and has the potential to improve our ability to tailor interventions.

Weaknesses

- One major limitation is that the biomedical intervention proposed for substance abuse is medication-assisted therapy (MAT), which is available for opioid use but not stimulant use. Besides alcohol and marijuana, the primary drugs use by MSM are stimulants (cocaine and methamphetamine), for which MAT is not available. Indeed, in the ASTART study, 40% tested positive for amphetamines and 30% for cocaine, but 0% for opioids.

4. Approach
Strengths

- The use of an evidence-based treatment as the comparison arm is a major strength of the proposal.
- The use of user-centered design to refine the uTech intervention is a strength.
- The use of responsive web design will allow for cross-platform compatibility and better security, thus increasing the potential for wide dissemination.
- The methods for the pilot testing and RCT are robust.
- The sample size is appropriate and supported by a power analysis.

Weaknesses

- It is disappointing that the project does not test the uptake of MAT or PrEP, given the focus on these biomedical interventions in the proposal.
- The goal of the proposed intervention is to increase uptake of PrEP and MAT. However, this presupposes that these services are readily available and affordable to the participants receiving the social media intervention, which they may not be. This should be addressed.
- As the US population becomes more and more concerned about privacy on social media sites, I am unclear how feasible it is to mine date generated on social medial sites, including the gathering of usernames and passwords. It appears that the PI was able to overcome privacy settings in his ASTART project by having participants sign consents indicating willingness. However, how will this be disseminated more widely in the community?
- A 57% response rate for weekly diaries is not impressive. Being able to validly capture sexual and substance use behavior throughout the study is important.
- I am concerned about the use of a previously developed machine learning algorithm. How stable is the algorithm over time? Will it get outdated and thus less effective at predicting who is at risk?
- The PI discusses alternate strategies considered in the development of uTech in his prior study but does not address potential challenges that might arise in the pilot and RCT phases of this proposal.

5. Environment
Strengths

- The environment at UCLA is excellent and provides sufficient resources to support the project.

Weaknesses

- None noted

Protections for Human Subjects: Acceptable Risks and/or Adequate Protections

Data and Safety Monitoring Plan (Applicable for Clinical Trials Only): Acceptable

Inclusion of Women, Minorities and Children Applicable Only for Human Subjects Research:

G3A - Only Men, Acceptable

M1A - Minority and Non-minority, Acceptable

C3A - No Children Included, Acceptable

Vertebrate Animals

- Not Applicable (No Vertebrate Animals)

Biohazards
- Not Applicable (No Biohazards)

Applications from Foreign Organizations
- Not Applicable (No Foreign Organizations)

Select Agents
- Not Applicable (No Select Agents)

Resource Sharing Plans

Unacceptable
- Not included

Budget and Period of Support: Recommend as Requested

Recommended budget modifications or possible overlap identified:

Additional Comments to Applicant (Optional):

CRITIQUE 3

Significance: 2
Investigator(s): 1
Innovation: 3
Approach: 4
Environment: 1

Overall Impact: Overall this project is a novel application of machine learning to reduce HIV transmission and treat opioid use disorders among gay, bisexual, and other men who have sex with men. The project proposes to develop *uTECH*, a biomedical prevention aimed at both HIV and substance use treatment and prevention, to examine the link between technology use and HIV risk behavior and to perform a feasibility trail to evaluate *uTECH*, as compared to an alternative intervention. The project is innovative in that the machine learning techniques will be developed using platforms already actively in use among the members of the population, which offer the potential for more scalable and cost-effective intervention delivery as compared to delivery by medical providers. In addition, the project aims to evaluate the implementation of *uTECH* among a sample of men who have sex with men. Dr. Holloway is well-suited for this project and has a successful funding and publication record in technology development based on social networking and substance use and HIV treatment and prevention among gay, bisexual, and other men who have sex with men. The application proposes to evaluate outcomes that are an important step in developing the intervention; however, patient outcomes, such as successful linkage to care, prevention of HIV and treatment of opioid use disorder, will not be evaluated, so the potential of the technology to have an impact on subsequent population health is not clear. The project addresses important barriers to reaching GBMSM substance users with

educational information for HIV prevention and treatment of substance use disorders and linkage to care by proposing a new machine learning technology tailored to the needs of this population.

1. Significance
Strengths

- The project addresses an important barrier to reaching GBMSM substance users with educational information for HIV prevention and treatment of substance use disorders by proposing a new machine learning technology tailored to the needs of this population.
- The project proposes to further evaluate the predictive power of social media data for health outcomes, which could prove to be an important and sustainable tool in improving health in hard-to-reach populations.
- The project identifies two interventions, PrEP and MAT, that are known to be effective, but underutilized, particularly among GBMSM, and proposes a novel methodology to increase the dissemination of information related to the two interventions, including how to access care.

Weaknesses

- Although the technology shows great promise for innovation, the proposed project does not evaluate the impact of technology on patient outcomes, including successful linkage to care or HIV risk reduction.
- The application mentions the integration of biomedical and behavioral intervention strategies but does not propose specific ways that the information about the two interventions, PrEP and MAT, or the interventions themselves could be successfully integrated.
- Although it is likely the *uTECH* is one intervention in a series of novel approaches, the proposed project does not describe how the work will lead to new HIV/AIDS interventions beyond *uTECH*.

2. Investigator(s)
Strengths

- The PI is an Associate Professor with Tenure at the University of California, Los Angeles. He has published more than 50 peer-reviewed manuscripts and won awards in the areas of preventing HIV transmission among gay, bisexual, and other men who have sex with me, alcohol and substance use, mental health problems, and factors that influence resilience.
- The PI developed the data collection and mining module that is the foundation for the *uTECH* program. The PI has demonstrated preliminary efficacy of machine learning models for among GBMSM who use substances, including a machine learning algorithm that helps participants understand their technology use in relation to HIV and substance use.
- The PI demonstrated the ability to pursue groundbreaking research through his work with geospatial technology use patterns and HIV risk. In addition, the PI has preliminary findings that patterns using natural language processing correlate with health behavior.

Weaknesses

- None noted

3. Innovation
Strengths

- This proposal is innovative because it leverages big data to personalize the intervention and integrates interventions aimed at HIV and substance use treatment and prevention among GBMSM substance users.

- The project will evaluate the acceptability, appropriateness, and feasibility of novel interventions as compared to existing interventions, an important step in scale-up of the technology.

Weaknesses

- The invention will deliver educational information, but no additional steps will be taken to successfully link participants to care, initiate treatment, or support participants to remain in care.
- The risks of this project beyond a traditional research project are not clearly described. If the project was funded under a traditional mechanism, the evaluation of important clinical endpoints among participants, such as reductions in condomless anal intercourse and substance use, could be evaluated.

4. Approach
Strengths

- The trial design includes through justification for the intervention groups and the trial is adequately powered for at least one outcome.
- The proposal includes a detailed description of the mediation and moderation analysis, as well as several proposed approaches that are appropriate in the analysis of the trial data. The trial design considers issues related to implementation of the intervention improving real-world impact.

Weaknesses

- The application does not include discussion of how to handle stigmatization issues around HIV and substance use. The information disseminated as part of the intervention could lead to stigmatization among members of the social network. Details on how stigmatization will be addressed are needed.
- Because a clinical trial is not allowed, the PI proposes a trial with process outcomes. However, no justification (beyond RFA requirements) is given for why the actual clinical outcomes are not of interest. In terms of patient outcomes, linkage to care for PrEP and MAT and retention in care would be of great interest and ultimately improve the health of the individual and their sexual network.
- The discussion of the implementation of machine learning included limited details. More details about the methodology that will be employed, including the strengths and limitations of these approaches, are needed.
- For the feasibility trial, the application does not discuss issues of selection bias in the analysis or generalizability of the trial results. Further justification for the rationale of *uTECH* being preferable over YMHP is needed. In addition, given the nature of the scientific question, a non-inferiority design may provide more power and better address the scientific question.
- There are some additional details needed for the analysis of the trial. More details are needed about how the missing values will be imputed. In addition, the baseline factors for adjustment should be specified in advance. Additional details are needed about how the time-varying mediators will be assessed.

5. Environment
Strengths

- The University of California Los Angeles includes the School of Public Affairs, Computer Science Department and School of Medicine, as well as centers for HIV/AIDS and gender-related issues. This environment with experts and resources in related fields will be supportive of this type of interdisciplinary project.
- The PI will have access to the Social Sciences Computing, which provides computing resources and a Secure Data Enclave, which will be important for the sensitive information collected as part of this project. The PI will also have access to the computing resources in the Department of Computer Science.

Weaknesses

- The application does not include details about how often the team will meet, including face-to-face meetings.

Protections for Human Subjects:

Unacceptable Risks and/or Inadequate Protections

- Depending on the level of geographic information, the data may be considered identifiable, so additional considerations may be required.

Data and Safety Monitoring Plan (Applicable for Clinical Trials Only): Unacceptable

  - More details are needed for the data and safety monitoring plan. Safety indictors should be developed and monitored to ensure participant safety.

Inclusion of Women, Minorities and Children Applicable Only for Human Subjects Research:

G3A - Only Men, Acceptable

M1A - Minority and Non-minority, Acceptable

C3A - No Children Included, Acceptable

Vertebrate Animals

- Not Applicable (No Vertebrate Animals)

Biohazards

- Not Applicable (No Biohazards)

Applications from Foreign Organizations

- Not Applicable (No Foreign Organizations)

Budget and Period of Support: Recommend as Requested

Recommended budget modifications or possible overlap identified:

Additional Comments to Applicant (Optional):

THE FOLLOWING SECTIONS WERE PREPARED BY THE SCIENTIFIC REVIEW OFFICER TO SUMMARIZE THE OUTCOME OF DISCUSSIONS OF THE REVIEW COMMITTEE, OR REVIEWERS' WRITTEN CRITIQUES, ON THE FOLLOWING ISSUES:

PROTECTION OF HUMAN SUBJECTS ACCEPTABLE.

INCLUSION OF WOMEN PLAN (Resume): ACCEPTABLE.

INCLUSION OF MINORITIES PLAN (Resume): ACCEPTABLE.

INCLUSION OF CHILDREN PLAN (Resume): ACCEPTABLE.

MEETING ROSTER

National Institute on Drug Abuse Special Emphasis Panel
NATIONAL INSTITUTE ON DRUG ABUSE
Avenir Award Program for Research on Substance Abuse and HIV/AIDS (DP2) applications

ZDA1 HXO-H (11)
11/30/2018 - 03/15/2019

Notice of NIH Policy to All Applicants: Meeting rosters are provided for information purposes only. Applicant investigators and institutional officials must not communicate directly with study section members about an application before or after the review. Failure to observe this policy will create a serious breach of integrity in the peer review process, and may lead to actions outlined in NOT-OD-14-073 at https://grants.nih.gov/grants/guide/notice-files/NOT-OD-14-073.html and NOT-OD-15-106 at https://grants.nih.gov/grants/guide/notice-files/NOT-OD-15-106.html, including removal of the application from immediate review.

CHAIRPERSON(S)

EL-BASSEL, NABILA, PHD
WILLMA AND ALBERT MUSHER PROFESSOR OF SOCIAL WORK
SOCIAL INTERVENTION GROUP
SCHOOL OF SOCIAL WORK
COLUMBIA UNIVERSITY
NEW YORK, NY 10025

MEMBERS

BUCHANAN, ASHLEY LYNN, DRPH
ASSISTANT PROFESSOR
THE UNIVERSITY OF RHODE ISLAND
COLLEGE OF PHARMACY
DEPARTMENT OF PHARMACY PRACTICE
KINGSTON, RI 02881

CHO, MICHAEL W, PHD
PROFESSOR
BIOMEDICAL SCIENCES
COLLEGE OF VETERINARY MEDICINE
IOWA STATE UNIVERSITY
AMES, IA 50011

COFFIN, PHILLIP O, MD
DIRECTOR OF SUBSTANCE USE RESEARCH
SAN FRANCISCO DEPARTMENT OF PUBLIC HEALTH
DIVISION OF HIV, ID AND GLOBAL HEALTH
UNIVERSITY OF CALIFORNIA, SAN FRANCISCO
SAN FRANCISCO, CA 94102

EISENBERG, MARISA C, PHD
ASSOCIATE PROFESSOR
DEPARTMENTS OF EPIDEMIOLOGY
COMPLEX SYSTEMS
AND MATHEMATICS UNIVERSITY OF MICHIGAN
ANN ARBOR, MI 48109

EUBANK, STEPHEN G, PHD
PROFESSOR
BIOCOMPLEXITY INSTITUTE & INITIATIVE
AND PUBLIC HEALTH SCIENCES
UNIVERSITY OF VIRGINIA
CHARLOTTESVILLE, VA 24060

GARAVAN, HUGH P, PHD
PROFESSOR
DEPARTMENT OF PSYCHIATRY
UNIVERSITY OF VERMONT
BURLINGTON, VT 05401

HEL, ZDENEK, PHD
PROFESSOR
DEPARTMENT OF PATHOLOGY
SCHOOL OF MEDICINE
UNIVERSITY OF ALABAMA AT BIRMINGHAM
BIRMINGHAM, AL 35294

JAIN, MAMTA K, MD
ASSOCIATE PROFESSOR
DEPARTMENT OF INTERNAL MEDICINE
UNIVERSITY OF TEXAS SOUTHWESTERN MEDICAL CENTER
DALLAS, TX 75390

LANDAU, NATHANIEL R., PHD
PROFESSOR
DEPARTMENT OF MICROBIOLOGY
SCHOOL OF MEDICINE
NEW YORK UNIVERSITY
NEW YORK, NY 10016

LINGWOOD, DANIEL, PHD
ASSISTANT PROFESSOR
DEPARTMENT OF MEDICINE
MASSACHUSETTS GENERAL HOSPITAL
HARVARD MEDICAL SCHOOL
CAMBRIDGE, MA 02139

MAGIDSON, JESSICA F, PHD
ASSISTANT PROFESSOR (CLINICAL)
DEPARTMENT OF PSYCHOLOGY
UNIVERSITY OF MARYLAND
COLLEGE PARK, MD 20742

MEADE, CHRISTINA S, PHD
ASSOCIATE PROFESSOR
DEPARTMENT OF PSYCHIATRY AND BEHAVIORAL SCIENCES
DUKE GLOBAL HEALTH INSTITUTE
SCHOOL OF MEDICINE
DUKE UNIVERSITY
DURHAM, NC 27708

MONTANER, LUIS J., DVM, PHD
KEAN FAMILY ENDOWED CHAIR PROFESSOR
DEPARTMENT OF IMMUNOLOGY
THE WISTAR INSTITUTE
PHILADELPHIA, PA 19104

NEWCOMB, MICHAEL E., PHD
ASSISTANT PROFESSOR
ASSISTANT PROFESSOR
DEPARTMENT OF MEDICAL SOCIAL SCIENCES
FEINBERG SCHOOL OF MEDICINE
NORTHWESTERN UNIVERSITY
CHICAGO, IL 60611

PAZGIER, MARZENA ELZBIETA, PHD
ASSOCIATE PROFESSOR
INFECTIOUS DISEASE DIVISION
DEPARTMENT OF MEDICINE (MED)
UNIFORMED SERVICES UNIVERSITY OF THE HEALTH SCIENCES
BETHESDA, MD 20814

PERUZZI, FRANCESCA, PHD
ASSOCIATE PROFESSOR
SECTION OF HEMATOLOGY/ONCOLOGY
DEPARTMENT OF INTERNAL MEDICINE
SCHOOL OF MEDICINE
LOUISIANA STATE UNIVERSITY
NEW ORLEANS, LA 70112

RAPPOCCIOLO, GIOVANNA, PHD
ASSISTANT PROFESSOR
UNIVERSITY OF PITTSBURGH
DEPARTMENT OF INFECTIOUS DISEASES AND MICROBIOLOGY
GRADUATE SCHOOL OF PUBLIC HEALTH
PITTSBURG, PA 15261

RUDOLPH, ABBY ELISE, PHD
ASSOCIATE PROFESSOR
COLLEGE OF PUBLIC HEALTH
TEMPLE UNIVERSITY
PHILADELPHIA, PA 19122

TSUI, JUDITH, MD
ASSOCIATE PROFESSOR OF MEDICINE
DIVISION OF GENERAL INTERNAL MEDICINE
UNIVERSITY OF WASHINGTON SCHOOL OF MEDICINE
HARBORVIEW MEDICAL CENTER
SEATTLE, WA 98122

WOODROW, KIM A., PHD
ASSOCIATE PROFESSOR
DEPARTMENT OF BIOENGINEERING
UNIVERSITY OF WASHINGTON
SEATTLE, WA 98122

SCIENTIFIC REVIEW OFFICER

ONO, HIROMI, PHD
SCIENTIFIC REVIEW OFFICER
OFFICE OF EXTRAMURAL AFFAIRS
NATIONAL INSTITUTE ON DRUG ABUSE, NIH, DHHS
BETHESDA, MD 20892

Consultants are required to absent themselves from the room during the review of any application if their presence would constitute or appear to constitute a conflict of interest.
